# Exosomes in cerebrospinal fluid as biomarkers of bacterial infections of the central nervous system: a pilot study

**DOI:** 10.3389/fcimb.2026.1760073

**Published:** 2026-02-11

**Authors:** Yunfeng Huang, Min Mei, Tianyu Fan, Jinfang Liu, Xin Li, Xin Chen, Shuai Zhang, Yong Gong, Peng Xiang, Jiangliu Yin

**Affiliations:** 1 Department of Neurosurgery, The Affiliated Changsha Central Hospital, Hengyang Medical School, University of South China, Changsha, China; 2 Department of Neurosurgery, Xiangya Neurosurgery, Xiangya Hospital, Central South University, Changsha, China; 3 Department of Neurosurgery, Hunan Brain Hospital (Hunan Second People’s Hospital), Changsha, China

**Keywords:** biomarkers, central nervous system, cerebrospinal fluid, exosomes, intracranial infection

## Abstract

**Introduction:**

Intracranial infections present a significant diagnostic challenge. This pilot study aimed to evaluate the potential of cerebrospinal fluid (CSF)-derived exosomes as novel diagnostic biomarkers for bacterial central nervous system infections.

**Methods:**

We enrolled nine patients from Changsha Central Hospital, forming three groups: culture-confirmed intracranial infection (n=3), cerebral hemorrhage with bloody CSF (n=3), and cerebral hemorrhage with clear CSF (n=3). Exosomes were isolated from CSF via ultracentrifugation and characterized using transmission electron microscopy and nanoparticle tracking analysis to assess morphology, concentration, and particle size. Statistical differences between groups were analyzed.

**Results:**

Exosomes were successfully detected in all but one clear-CSF patient. The infected CSF group exhibited a significantly higher mean exosome concentration (1.37e+11 ± 4.40e+10 particles/ml) compared to the bloody (4.08e+9 ± 1.95e+9 particles/ml) and clear CSF groups (1.10e+9 ± 0.56e+9 particles/ml; both p<0.001). Exosome diameter was also significantly larger in infected CSF (167.78 ± 18.65 nm) than in bloody (146.63 ± 6.92 nm) and clear CSF (123.77 ± 13.68 nm; both p<0.001).

**Conclusions:**

This study demonstrates that both the concentration and size of CSF exosomes are significantly elevated in patients with intracranial infections. These findings suggest CSF exosomes hold promise as valuable diagnostic biomarkers, though validation in larger cohorts is necessary to confirm their clinical utility.

## 1 Introduction

Central nervous system (CNS) infections are caused by a broad spectrum of pathogens, including bacteria, viruses, fungi, and parasites (Giovane and Lavender, 2018). These infections can involve various CNS compartments, such as the meninges (meningitis), brain parenchyma (encephalitis), and spinal cord (myelitis) (Ippolito et al., 2022). Severe CNS infections are often life-threatening and can result in permanent neurological disability (Bloch and Hasbun, 2021). Early and accurate diagnosis is critical for improving clinical outcomes; however, current diagnostic methods, particularly in emergency settings, often lack adequate sensitivity and specificity (Masouris et al., 2020). Therefore, there is an urgent need for additional diagnostic biomarkers.

Definitive diagnosis typically relies on direct pathogen detection via cerebrospinal fluid (CSF) culture or metagenomic next-generation sequencing (mNGS), or on the identification of pathogen-specific antibodies. These approaches, however, have practical limitations, including low positive rates, long turnaround times, and reduced diagnostic accuracy in patients who have received prior antimicrobial therapy (Masouris et al., 2020). Conventional CSF parameters, such as white blood cell counts and differentials, CSF-to-serum albumin and glucose ratios, chloride levels, and lactate concentrations, may provide indirect diagnostic clues but are insufficient for guiding timely treatment (Wiegand et al., 2016). Consequently, positive CSF culture remains the gold standard for bacterial meningitis and ventriculitis, despite its well-recognized shortcomings.

To address these challenges, research has increasingly focused on identifying novel CSF biomarkers that could enable early and reliable diagnosis of CNS infections. Among these, exosomes have emerged as promising candidates. Exosomes are small extracellular vesicles, typically 40–160 nm in diameter, actively secreted by cells of endosomal origin (Chiasserini et al., 2014). They play a key role in intercellular communication and carry diverse biomolecules, including proteins, mRNA, and microRNAs, which can be isolated from CSF (Budnik et al., 2016). Advances in exosome isolation techniques now allow proteomic or nucleic acid analyses from minimal CSF volumes (Chiasserini et al., 2014; Pulliam et al., 2019). Importantly, exosomes are implicated in neuroinflammation, CNS repair, and protein aggregation in neurological disorders such as multiple sclerosis, Alzheimer’s disease, and Parkinson’s disease (Vanherle et al., 2020). While viral CNS infections, including HIV-1, HTLV-1, and EBV—have been shown to alter host exosome composition and function (Sampey et al., 2014; Luong and Olson, 2021), the role of exosomes in bacterial CNS infections remains underexplored.

Building on this background, the present pilot study aimed to explore the potential diagnostic value of CSF-derived exosomes in patients with intracranial infections.

## 2 Materials and methods

### 2.1 Ethics

This study was approved by the Ethics Committee of Changsha Central Hospital (Approval No.20190709), and written informed consent was obtained from all patients or their legal representatives. The study was conducted in accordance with the ethical standards laid down in the 1964 Declaration of Helsinki.

### 2.2 Patients and controls

This pilot study was conducted at Changsha Central Hospital, a 3,000-bed tertiary-care medical center. Patients with confirmed intracranial infections based on CSF culture were enrolled in the experimental group. The control group comprised patients with cerebral hemorrhage, intracranial aneurysm, or cerebral trauma without evidence of intracranial infection. Based on the macroscopic appearance of CSF, the control group was further subdivided into bloody CSF and clear CSF subgroups.

### 2.3 Clinical data collection

Demographic and clinical data were retrieved from electronic medical records, including age, sex, primary neurological diagnosis, and comorbidities. CSF routine and biochemical indices were collected, including glucose, chloride, total protein, total cell counts, white blood cell counts, and the ratio of mononuclear to multinuclear cells. Microbiological results, including CSF culture findings and antimicrobial susceptibility, were recorded. Detailed antibiotic regimens, comprising drug name, dosage, frequency, and administration route, were also documented. Clinical outcomes at hospital discharge were categorized as cure, improvement, or treatment abandonment.

### 2.4 Microbiology

Bacterial susceptibility to antimicrobial agents was determined using the broth microdilution method with the VITEK^®^2 system (bioMérieux, Marcy-l’Étoile, France). Minimum inhibitory concentrations (MICs) were interpreted according to the guidelines of the European Committee on Antimicrobial Susceptibility Testing (EUCAST) and the Clinical and Laboratory Standards Institute (CLSI) guidelines (Dalyan et al., 2018). “Carbapenem resistance” was defined as strains isolated with non-susceptibility (MIC ≥4 mg/L) to imipenem or meropenem, according to the CLSI 2019 criteria (Dalyan et al., 2018). Resistance to polymyxins and tigecycline was defined as MIC >2 mg/L, following EUCAST guideline (http://www.eucast.org/clinical_breakpoints) (Dalyan et al., 2018).

### 2.5 Extraction and characterization of CSF exosomes

After infection confirmation, patients in the intracranial infection group provided CSF for microbiological culture and exosome analysis. Control patients donated CSF during routine drainage, either with bloody or clear appearance. Exosomes were isolated using classical differential ultracentrifugation, a widely accepted standard for extracellular vesicle purification (Kowal et al., 2017). Due to limited CSF volumes, no additional concentration or ultrafiltration steps were applied (Sandau et al., 2024). Briefly, CSF samples were centrifuged sequentially as follows: 300g for 10 minutes at 4°C, 2000g for 30 minutes, 12000g for 45 minutes, and finally 110000g for 120 minutes. The resulting pellet was resuspended in 1500ul of PBS to obtain purified exosomes, which were stored at -80°C.

### 2.6 Cerebrospinal fluid extracellular vesicles

For morphological analysis, exosomes were fixed with glutaraldehyde, placed dropwise onto copper grids, and negatively stained with 2% phosphotungstic acid for 2 minutes at room temperature. Specimens were examined using transmission electron microscopy (TEM, G2 Spirit FEI, USA). Nanoparticle tracking analysis (NTA) was performed to assess particle size distribution and concentration, with vesicle Brownian motion monitored to calculate hydrodynamic diameter and particle concentration using the Stokes–Einstein equation.

### 2.7 Statistical analysis

Statistical analyses were conducted using SPSS 29.0 (IBM, Armonk, USA). Continuous variables were expressed as mean ± standard deviation (SD) for normally distributed data, or as median with interquartile range (IQR) for skewed distributed data. Between-group comparisons were performed using one-way analysis of variance (ANOVA) for normally distributed variables, or nonparametric tests for skewed data. The Kruskal–Wallis test was applied for multiple-group comparisons, with pairwise analyses conducted when significant differences were observed.

## 3 Results

### 3.1 Study population and clinical characteristics

A total of nine patients were included in this pilot study: three with culture-confirmed intracranial infections, three with cerebral hemorrhage and bloody CSF, and three with cerebral hemorrhage and clear CSF. Baseline demographic and clinical characteristics of all participants are summarized in **Table 1**. The pathogens isolated from infected CSF were Escherichia coli, Acinetobacter baumannii, and Enterobacter cloacae. Antimicrobial susceptibility testing revealed carbapenem resistance in A. baumannii, which remained sensitive only to polymyxins and tigecycline. In contrast, E. coli and E. cloacae isolates were susceptible to carbapenems (**Table 2**).

**Table 1:** The clinical characteristics of enrolled samples.

Patients number	Subgroup	Sex	Age (years)	Diagnosis	CSF			CSF		CSF culture		Antibiotic use	Clinical efficacy
					Glu(mmol/l)	Cl(mmol/l)	TP(mg/l)	Total cell counts(*10^6^)	WBC count(*10^6^)	Mononuclear cells/multiple nuclear cells			
1	Infection	male	55	Cerebral trauma, intracranial infection	0.27	115.2	3.93	36800	36400	5% vs 95%	Eescherichia coli	MER 2.0g, q8h+ LZD 600mg,q12h+ AMK 20mg Intrathecal injection	cure
2	Infection	male	52	Cerebral trauma, intracranial infection	0.1	118.7	4.04	6000	5600	10% vs 90%	Acinetobacter baumannii	MER 2.0g, q8h, PMB100mg, q12h + 5mg Intrathecal injection	cure
3	Infection	male	64	Cerebral hemorrhage, intracranial infection	1.42	123.4	2.67	4055	55	10% vs 90%	Enterobacter cloacae	MER 2.0g, q8h+ VAN 1.0g, q12h	improve
4	Bloody cerebrospinal fluid	female	70	Intracranial aneurysm	6.22	115	2.12	2691	185	59% vs 41%	NA	CPM 2.0g, q8h	improve
5	Bloody cerebrospinal fluid	male	65	Cerebral trauma, Cerebral hemorrhage	2.44	142	2.07	24200	200	40% vs 60%	NA	CPM 2.0g, q8h	improve
6	Bloody cerebrospinal fluid	female	58	Cerebral hemorrhage	1.8	135	2.81	10680	1680	85% vs. 15%	NA	CPM 2.0g, q8h	give up
7	Clear cerebrospinal fluid	male	58	Intracranial aneurysm	4.53	120	0.91	18	10	80% vs. 20%	NA	TZP 4.5g, q8h	cure
8	Clear cerebrospinal fluid	male	74	Cerebral hemorrhage	6.42	128	0.63	1	1	95% vs. 5%	NA	TZP 4.5g, q8h	improve
9	Clear cerebrospinal fluid	male	66	Cerebral hemorrhage	4.57	119	0.85	33	30	76% vs 24%	NA	CAZ 2.0g, q8h	improve

MER, meropenem; TZP, piperacillin/tazobactam; LZD, linezolid; CPM, cefepime; VAN, vancomycin; AMK, amikacin; Glu, glucose; Cl, chlorine; TP, total protein; NA, negative.

**Table 2:** The antimicrobial susceptibility test of bacteria.

Antibiotic	*Acinetobacter baumannii*			*Escherichia coli*			*Enterobacter cloacae*		
	Result	Sensitivity	Reference	Result	Sensitivity	Reference	Result	Sensitivity	Reference
Piperacilin/Tazobactam	≥128	R	S ≤ 16 R≥128	≤4	S	S ≤ 16 R≥128	≤8	S	S ≤ 8/4 R≥32/4
Cefoperazone/Sulbactam	≥64	R	S ≤ 16 R≥64	25	S	S≥21 R ≤ 15	≤16	S	S ≤ 16 R≥64
Ampicillin/Sulbactam	6	R	S≥15 R ≤ 11	17	S	S≥15 R ≤ 11	≥32	R	
Ceftazidime	≥64	R	S ≤ 8 R≥32				16	R	S ≤ 16 R≥64
Cefepime	≥32	R	S ≤ 8 R≥32	≤1	S	S ≤ 2 R≥16	4	I	S ≤ 2 R≥16
Aztreonam				≤1	S	S ≤ 4 R≥16	≥16	R	S ≤ 4 R≥16
Imipenem	≥16	R	S ≤ 2 R≥8	≤1	S	S ≤ 1 R≥4			
Meropenem	≥16	R	S ≤ 2 R≥8	30	S	S≥23 R ≤ 19	≤1	S	S ≤ 1 R≥4
Amikacin	6	R	S≥17 R ≤ 14	≤2	S	S ≤ 16 R≥64	≤8	S	S ≤ 16 R≥64
Ciprofloxacin	≥4	R	S ≤ 1 R≥4	≤0.25	S	S ≤ 0.25 R≥1			
Levofloxacin	≥8	R	S ≤ 2 R≥8	≤0.25	S	S ≤ 0.5 R≥2	25	S	S ≤ 0.5 R≥2
Sulfamethoxazole	≥16/304	R	S ≤ 2 R≥4	≥16/304	R	S ≤ 2 R≥4	≥8	R	S ≤ 2 R≥4
Polymyxin	≤0.5	S	S ≤ 2 R≥4				≤1	S	S ≤ 2 R≥4
Minocycline	≥16	R	S ≤ 4 R≥16				≤4	R	S ≤ 4 R≥16
Tigecycline	2	S	S ≤ 2 R≥8	≤0.5	S	S ≤ 2 R≥8	≤1	S	S ≤ 2 R≥8
Piperacilin	6	R	S≥21 R ≤ 17						
Amoxicillin/Clavulanate				8	S	S ≤ 8 R≥32			
Cefazolin				≥64	R	S ≤ 2 R≥8	≥32	R	S ≤ 2 R≥8
Ceftriaxone				≥64	R	S ≤ 1 R≥4	≥64	R	
Cefoxitin				≤4	S	S ≤ 8 R≥32	16	R	S ≤ 8 R≥32
Ampicillin				≥32	R	S ≤ 8 R≥32	≥32	R	
Ertapenem				≤0.5	S	S ≤ 0.5 R≥2			
Cefuroxime				6	R	S≥18 R ≤ 14	≥32	R	S ≤ 8 R≥32

R, resistant; S, sensitive.

### 3.2 Isolation and characterization of exosomes

**Figure 1:**
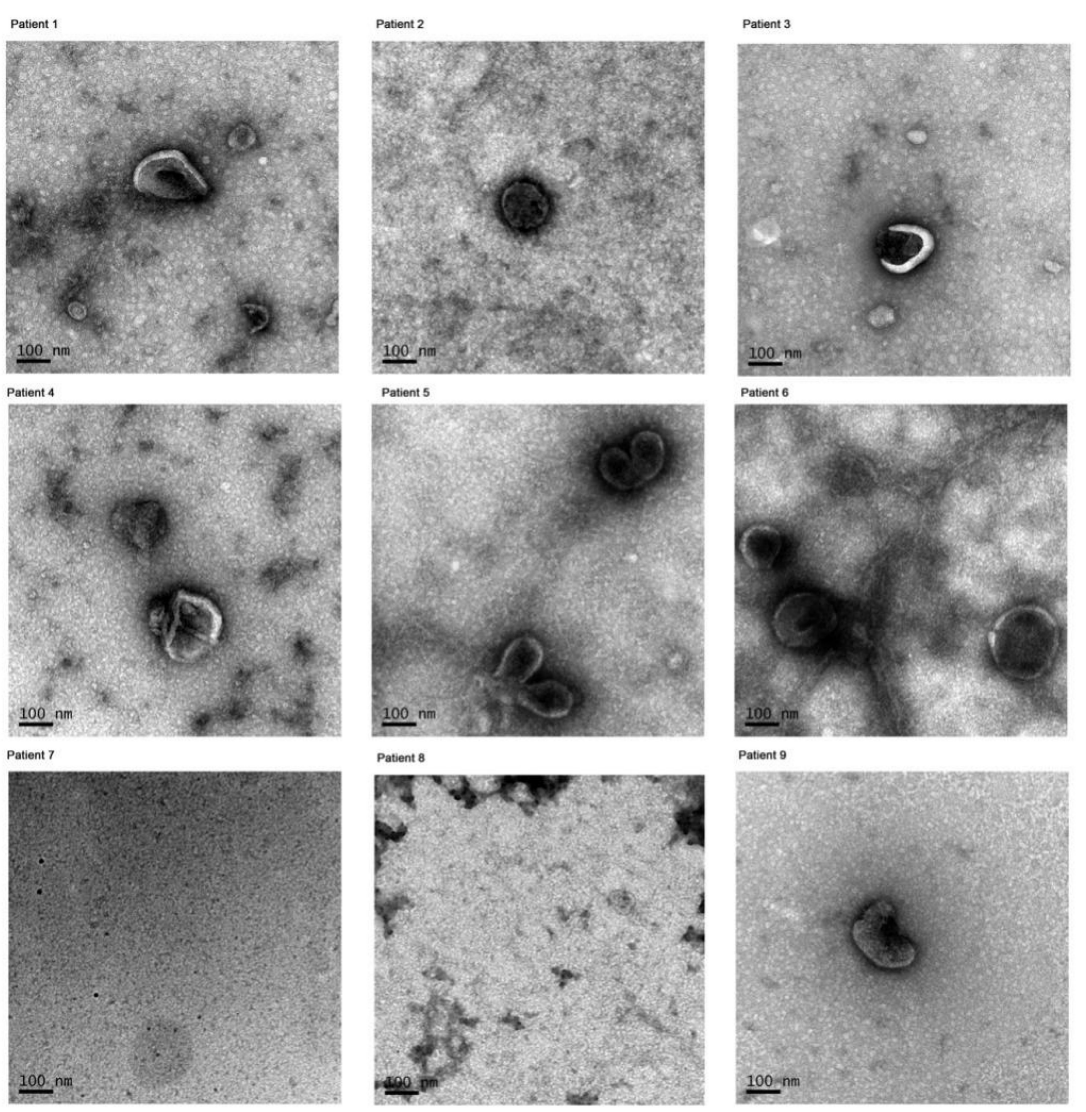
Morphologies of exosomes were observed using TEM in different patients. Patient 1–3 with intracranial infection, patient 4–6 with cerebral hemorrhage and bloody CSF, patient 7–9 with cerebral hemorrhage and clear CSF.

Exosome-like vesicles were successfully isolated from the CSF of all patients, except for one case with clear CSF secondary to cerebral hemorrhage (Patient 8). TEM imaging confirmed the presence of round, membrane-bound vesicles consistent with exosome morphology (**Figure 1**).

### 3.3 Exosome concentration and particle size

**Figure 2:**
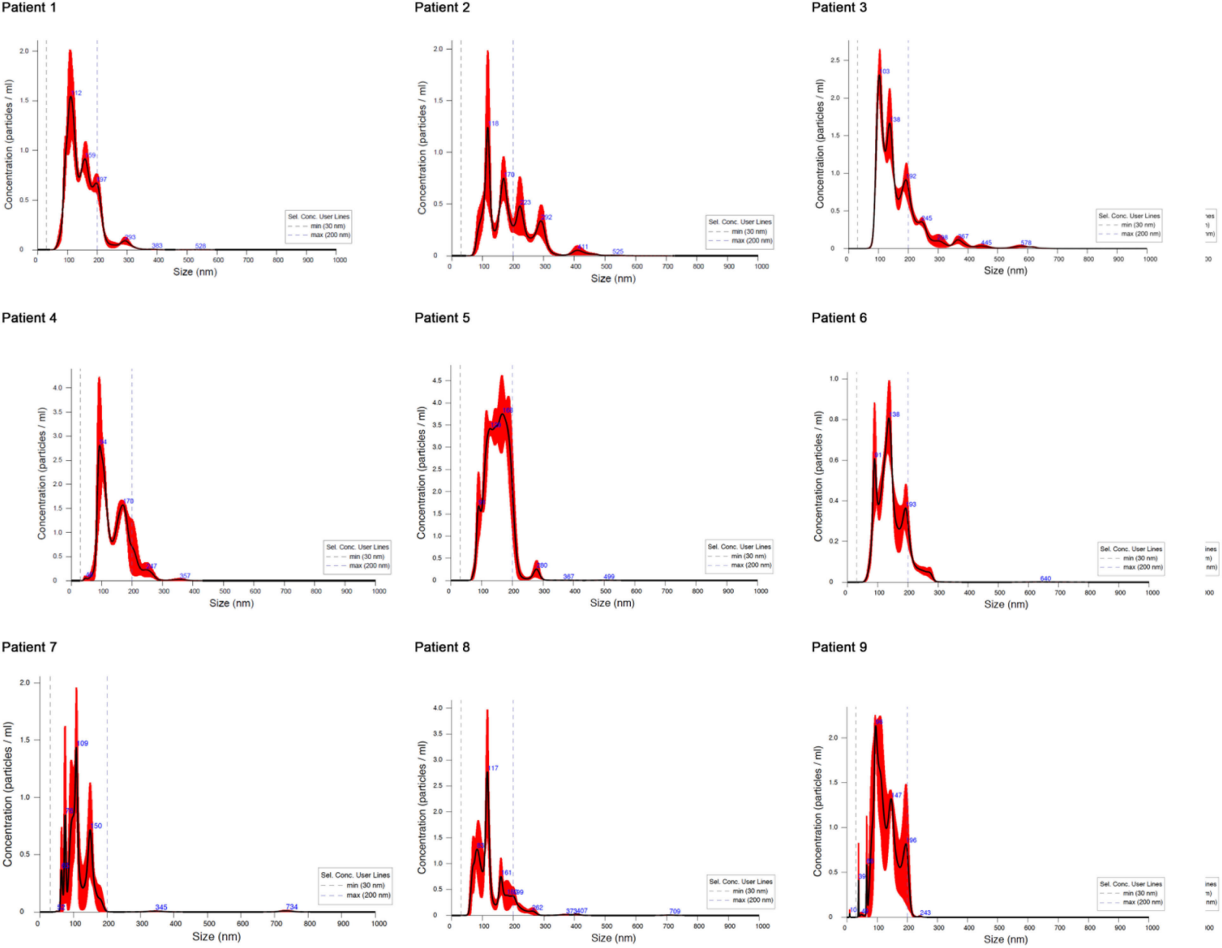
The size of exosomes isolated from CSF in different patients. Patient 1–3 with intracranial infection, patient 4-6 with cerebral hemorrhage and bloody CSF, patient 7–9 with cerebral hemorrhage and clear CSF.

Quantitative analysis revealed significant differences in exosome concentration among the three groups (**Table 3**; **Figure 2**). The mean concentration in infected CSF was significantly higher (1.37e+11 ± 4.40e+10 particles/ml) compared with both bloody CSF (4.08e+9 ± 1.95e+9 particles/ml) and clear CSF (1.10e+9 ± 0.56e+9 particles/ml) (both p < 0.001).

**Table 3:** The concentration/size of exosomes isolated from CSF.

Patients number	Subgroup	Group average concentration (particles/ml)	Concentration (particles/ml)	Concentration p value	Average particle size (nm)	Particle concentration within a distribution range of 30-200nm (particles/ml) (percentage of totals)	Particle size p value
1	Infection	1.37e+11 ± 4.40e+10	1.27e+11 ± 7.63e+09	reference	148.7 ± 3.5	1.10 E + 11 (86.2%)	reference
2	Infection		9.76e+10 ± 1.99e+10		186.2 ± 8.4	6.11 E + 10 (62.6%)	
3	Infection		1.86e+11 ± 1.10e+10		168.4 ± 5.4	1.43 E + 11 (77.0%)	
4	Bloody cerebrospinal fluid	4.08e+9 ± 1.95e+9	2.04e+09 ± 4.08e+08	<0.001	142.4 ± 4.4	1.79 E + 9 (87.8%)	<0.001
5	Bloody cerebrospinal fluid		3.84e+09 ± 2.04e+08		152.9 ± 1.2	3.53 E + 9 (92%)	
6	Bloody cerebrospinal fluid		6.36e+09 ± 4.18e+08		144.6 ± 3.6	5.63 E + 9 (88.4%)	
7	Clear cerebrospinal fluid	1.10e+9 ± 0.56e+9	5.76e+08 ± 1.24e+08	<0.001	116.9 ± 9.5	5.68 E + 8 (98.7%)	<0.001
8	Clear cerebrospinal fluid		1.26e+09 ± 1.56e+08		129.4 ± 6.5	1.14 E + 9 (90.3%)	
9	Clear cerebrospinal fluid		1.47e+09 ± 4.00e+08		125.1 ± 8.7	1.41 E + 9 (96%)	

Exosome particle size also differed significantly among groups (F = 22.44, p < 0.001). Exosomes in infected CSF were larger on average (167.78 ± 18.65 nm) than those in bloody CSF (146.63 ± 6.92 nm) and clear CSF (123.77 ± 13.68 nm) (both p < 0.001). The distribution of particle sizes within the 30–200 nm range is detailed in **Table 3**.

## 4 Discussion

Intracranial infection poses a significant diagnostic challenge in clinical practice, as current methods often lack sufficient sensitivity and specificity, particularly in emergency settings (Masouris et al., 2020). Exosomes and other extracellular vesicles (EVs) are nanoscale particles (50–200 nm in diameter) secreted by nearly all cell types and detectable in various body fluids, including urine, plasma, and CSF (Chakrabortty et al., 2022). Previous studies have shown that viruses can exploit exosomes to facilitate infection, whereas bacteria can release vesicles that damage host cells (Kalluri and LeBleu, 2020). Exosomes are also recognized as key mediators of immune and inflammatory responses during infectious processes (Rivera et al., 2010; Goncalves et al., 2023). However, the role of exosomes in bacterial CNS infections remains poorly understood. In this pilot study, we investigated whether CSF-derived exosomes could serve as diagnostic indicators of intracranial infection. Exosomes were successfully isolated and characterized from nine CSF samples, comprising three culture-confirmed intracranial infections and six controls. Quantitative analysis revealed significantly higher concentrations and larger particle sizes of exosomes in infected CSF compared with both bloody and clear CSF controls. These findings suggest that CSF exosomes may hold promise as potential biomarkers for intracranial infections.

Recently, exosomes and other EVs have garnered increasing attention in the study of neuroinfectious diseases. These nanoscale vesicles, actively secreted by cells and present in virtually all body fluids, are increasingly recognized as critical mediators of intercellular communication and immune responses (Budnik et al., 2016; Pulliam et al., 2019). Viruses can hijack exosomal pathways to promote infection, while bacterial vesicles can contribute to host cell damage. The capacity of exosomes to encapsulate diverse molecular cargo, including proteins, nucleic acids, and metabolites, makes them a promising source of diagnostic and prognostic biomarkers (Xie et al., 2023). Nonetheless, their involvement in bacterial CNS infections remains insufficiently explored. In this pilot study, we demonstrate that exosome concentration and particle size in CSF are elevated during intracranial infection, providing preliminary evidence that these features may serve as measurable indicators for diagnostic purposes.

The clinical implications of these findings are substantial. Current diagnostic approaches often delay therapeutic decision-making, particularly in patients with suspected bacterial meningitis or post-neurosurgical ventriculitis (Hussein et al., 2017). In contrast, exosome-based assays have the potential to deliver rapid, non-culture-based evidence of infection, thereby enabling earlier initiation of targeted therapy (Zhang et al., 2020). In neurosurgical practice, where diagnostic delays can lead to severe outcomes such as hydrocephalus, cerebral edema, or irreversible neurological deficits (Winkler et al., 2016), the detection of exosome signatures may serve as a valuable adjunct to existing diagnostic algorithms.

A methodological strength of this study lies in the combined application of TEM and NTA for exosome characterization. TEM allows direct visualization of the characteristic cup-shaped or spherical vesicular morphology at high resolution, confirming the structural integrity of isolated exosomes (Akhtar and Zuhair, 2025). NTA, in turn, provides quantitative information on particle size distribution and concentration based on Brownian motion (van der Pol et al., 2014). The integration of these complementary techniques not only enhances the reliability and accuracy of exosome identification but also strengthens the translational relevance of our findings.

The observed increase in exosome concentration and size in infected CSF likely reflects their heterogeneous origin. Exosomes may be released from host immune or neural cells under pathological stress or may include bacterial extracellular vesicles (BEVs) containing lipopolysaccharides, proteins, nucleic acids, and virulence factors (Chronopoulos and Kalluri, 2020). Previous studies have demonstrated that both viral and bacterial infections alter extracellular vesicle release and composition, subsequently modulating host immune responses (Lee et al., 2018; Toyofuku et al., 2019). Our results align with this body of evidence, supporting the notion that CSF in infected states exhibits a distinct exosomal profile in both quantity and size distribution. The larger particle size observed in infected CSF may also hold biological significance. Exosome size heterogeneity can arise from variations in membrane invagination during vesicle formation, resulting in differential cargo loading (Phillips et al., 2021). During infection, vesicles may encapsulate increased amounts of inflammatory mediators, bacterial components, or host-derived proteins, which could account for the observed increase in average size (Taylor and Gercel-Taylor, 2014). These alterations not only provide diagnostic insights but may also illuminate the underlying pathophysiological mechanisms of intracranial infections.

In addition to bacterial infections, previous studies have reported that viral and fungal CNS infections also influence exosome release and composition. For example, viral infections such as HIV-1 and EBV can incorporate viral proteins or RNA into host exosomes, modulating immune responses in uninfected cells, while limited evidence suggests that fungal pathogens may induce EV-mediated signaling to influence host-pathogen interactions (Caobi et al., 2020; Saad et al., 2021). Compared with these infection types, bacterial CNS infections appear to induce distinct exosomal profiles, reflected in both particle concentration and size, which may carry pathogen-associated molecular patterns (PAMPs) as well as host-derived inflammatory mediators (Mirzaei et al., 2021). By highlighting these differences, our study emphasizes the potential specificity of CSF exosomes as biomarkers for bacterial intracranial infections. These comparisons with viral and fungal infections underscore the innovative aspect of the present study. While prior research has largely focused on exosomes in viral CNS disease or systemic infections, few studies have characterized exosomes in the context of bacterial CNS infections, particularly in post-neurosurgical patients (Heidari et al., 2023). Our findings provide preliminary evidence that exosome concentration and size distribution in CSF may serve as measurable indicators that are distinct from other infection types, suggesting a unique diagnostic and pathophysiological role for exosomes in bacterial intracranial infections.

Proteomic studies further underscore the relevance of exosomes in CNS infections. In bacterial meningitis, for instance, upregulated exosome-associated proteins have been linked to inflammatory and antibacterial responses, suggesting that specific protein signatures could distinguish infectious from non-infectious etiologies (Peng et al., 2023). Likewise, in viral CNS infections such as HIV-1 or EBV, viral particles or viral RNA can be incorporated into exosomes, modulating immune activation in bystander cells (Khan and Ahmed, 2017; Ouattara et al., 2018). By extension, bacterial infections may induce the release of exosomes enriched with PAMPs and host-derived danger signals, thereby amplifying inflammatory cascades within the CNS (Adamczyk et al., 2023). Clinically, these findings highlight the potential of CSF exosomes as rapid biomarkers for differentiating infected from non-infected patients. This distinction is particularly critical in postoperative neurosurgical populations, where fever and altered consciousness may arise from diverse etiologies, and misdiagnosis can result in either unnecessary antibiotic exposure or harmful delays in treatment. Integration of exosome analysis into standard CSF testing workflows could therefore enhance diagnostic accuracy, reduce decision-making time, and optimize antimicrobial stewardship.

Several challenges must be addressed before clinical implementation. First, standardization of exosome isolation and characterization methods is essential. Although ultracentrifugation combined with TEM and NTA is widely employed, these techniques are time-intensive and technically demanding. Development of rapid, clinically applicable platforms for exosome detection will be crucial to translating these findings into practice (Helwa et al., 2017). Second, comprehensive molecular profiling of exosomal cargo—encompassing proteomics, transcriptomics, and lipidomics—will be necessary to identify infection-specific signatures. Such markers could not only support diagnosis but also enable stratification of disease severity and prediction of treatment response (Kugeratski et al., 2021).

This study has several limitations. The sample size was small, reflecting its pilot nature, which constrained statistical power and increased susceptibility to random bias. Characterization was limited to morphological and quantitative analyses, without in-depth assessment of exosomal cargo or functional roles. We did not distinguish between host-derived vesicles and bacterial extracellular vesicles, an issue requiring further mechanistic investigation. Additionally, as a single-center study, the generalizability of our findings may be limited. Importantly, we did not analyze molecular markers within exosomes, such as glial fibrillary acidic protein (GFAP), ubiquitin carboxy-terminal hydrolase L1 (UCH-L1), or neurofilament light chain (NFL), which correlate strongly with brain injury. The absence of these markers limits exploration of the relationship between exosomal content and underlying neuropathology. In addition, the origin of the isolated vesicles could not be definitively determined in this study. Although exosome-like vesicles were identified by TEM and NTA, molecular markers of host-derived exosomes or bacterial extracellular vesicles were not assessed. Thus, the contribution of bacterial vesicles in infected CSF cannot be excluded. Also, this study did not investigate the biological functions of infection-related exosomes. No *in vitro* cell-based experiments were performed to assess the effects of CSF-derived exosomes on microglia or astrocytes, nor to evaluate their influence on inflammatory cytokine expression. Therefore, the potential immunomodulatory roles of exosomes in CNS infections remain to be clarified and warrant further mechanistic studies. Future research should therefore aim to expand sample size, validate these preliminary observations in multicenter cohorts, incorporate molecular characterization to distinguish vesicle sources, and apply advanced omics approaches to dissect exosome composition. Parallel *in vitro* and *in vivo* studies will be essential to elucidate the precise roles of exosomes in the pathogenesis of intracranial infections. Ultimately, combining exosome analysis with conventional diagnostic methods could establish a multimodal approach, improving both sensitivity and specificity in CNS infection detection.

In addition, despite the promising findings, several limitations must be considered before CSF exosomes can be applied as routine diagnostic biomarkers. First, the isolation and characterization methods, including ultracentrifugation, TEM, and NTA, are technically demanding and time-consuming, which may hinder rapid clinical implementation (Lai et al., 2022). Second, exosome integrity and composition can be affected by sample handling and storage conditions, such as freeze-thaw cycles or prolonged preservation, potentially influencing quantitative and qualitative results (Lee et al., 2025). Third, CSF exosome levels may be altered by other inflammatory or neurological conditions, including traumatic brain injury or systemic infections, which could reduce diagnostic specificity (Guedes et al., 2020). These factors underscore the need for standardized protocols and larger studies to validate the reliability and clinical utility of exosome-based diagnostics.

## 5 Conclusion

In summary, this study provides early evidence that CSF exosome concentration and size differ significantly between infected and non-infected patients. These findings underscore the potential of exosomes as clinically valuable biomarkers for the diagnosis of intracranial infections, particularly in neurosurgical practice where rapid, accurate decision-making is essential. Although larger and more comprehensive studies are needed, the results of this pilot investigation lay the groundwork for future exploration of exosome-based diagnostics in CNS infectious diseases.

## Data Availability

The original contributions presented in the study are included in the article/supplementary material. Further inquiries can be directed to the corresponding author.
